# Host-Related Factors in the Interplay among Inflammation, Immunity and Dormancy in Breast Cancer Recurrence and Prognosis: An Overview for Clinicians

**DOI:** 10.3390/ijms24054974

**Published:** 2023-03-04

**Authors:** Lorenzo Ruggieri, Anna Moretti, Rossana Berardi, Maria Silvia Cona, Davide Dalu, Cecilia Villa, Davide Chizzoniti, Sheila Piva, Anna Gambaro, Nicla La Verde

**Affiliations:** 1 Medical Oncology Unit, Luigi Sacco University Hospital, ASST Fatebenefratelli-Sacco, Via G.B. Grassi, n° 74, 20157 Milan, Italy; 2 Medical Oncology Unit, S. Carlo Hospital, ASST Santi Paolo e Carlo, 20153 Milan, Italy; 3 Department of Oncology, Università Politecnica delle Marche—AOU delle Marche, 60121 Ancona, Italy; 4 Medical Oncology Unit, Fatebenefratelli Hospital, ASST Fatebenefratelli-Sacco, 20157 Milan, Italy

**Keywords:** dormancy, dormant, breast cancer, inflammation, immune escape, host-related factors, lifestyle

## Abstract

A significant proportion of patients treated for early breast cancer develop medium-term and late distant recurrence. The delayed manifestation of metastatic disease is defined as “dormancy”. This model describes the aspects of the clinical latency of isolated metastatic cancer cells. Dormancy is regulated by extremely complex interactions between disseminated cancer cells and the microenvironment where they reside, the latter in turn influenced directly by the host. Among these entangled mechanisms, inflammation and immunity may play leading roles. This review is divided into two parts: the first describes the biological underpinnings of cancer dormancy and the role of the immune response, in particular, for breast cancer; the second provides an overview of the host-related factors that may influence systemic inflammation and immune response, subsequently impacting the dynamics of breast cancer dormancy. The aim of this review is to provide physicians and medical oncologists a useful tool to understand the clinical implications of this relevant topic.

## 1. Introduction

Breast cancer (BC) is the most common type of cancer in women and represents one of the most important concerns for global health [1,2]. A significant proportion of patients experience distant recurrence despite having received curative treatment for early-stage disease. Tumor characteristics (such as stage at diagnosis, biological subtype and site of metastasis) and patient characteristics (such as age, body mass index (BMI) and menopausal status) mainly determine the risk of distant recurrence and pattern of relapse [3,4]. Late distant relapse is a specific characteristic of BC, occurring even 20 years after the definitive treatment for localized or locally advanced disease [5,6]. In this regard, the improvement in the risk of recurrence and cancer-specific mortality due to the extension of adjuvant endocrine therapy in hormone-positive BC is probably related to the prolongation of the interval of metastatic latency [7,8]. 

To date, the biological basis of clinical latency in BC is poorly understood. Metastatization appears to occur early during primitive BC development, since disseminated cancer cells isolated from the bone marrow of patients treated for early breast cancer display less advanced genomic features compared to primitive tumor cells [9,10]. Once the metastatic subclones reach the different organs, they indwell in specific tissue sites, known as niches, remaining undetectable for variable periods before resuming proliferation and, subsequently, manifesting themselves as visible disease. These specific behaviors of metastatic disease are synthesized as “dormancy”. This is a speculative model used to explain the inherent biology of latent metastatic disease. This model aims to explain the specific interactions that occur between cancer cells, niche-resident cells and the immune system. The model of dormancy hypothesized that, after curative treatment for early breast cancer, single isolated cells persist as minimal residual metastatic disease and remain quiescent for a specific period. These cells, known as dormant cancer cells (DCCs), spread alongside the body and hide in specific tissue spots known as metastatic niches. Here, DCCs resist the cytotoxic effect of chemotherapy due to both their quiescent status and the active protection conferred by the niches [11,12,13]. In the metastatic niches, DCCs actively interact with resident and immune-competent cells, creating a favorable microenvironment where they survive and maintain a long-lasting equilibrium with the host, sometimes lasting for decades [6,14,15,16]. Certain niches are organ-specific, such as the endosteal niche in the bone marrow; others, such as the perivascular niche, are common to different organs (brain, lung, bone, etc.). Additionally, DCCs are able to reawaken from dormancy, restart their proliferation cycle and potentially lead to subsequent distant relapse [16]. The ability to switch proliferation mechanisms on and off, to persist even during antiproliferative therapy and to escape the immune response represent crucial mechanisms for DCC survival [14]. Essentially, these specific mechanisms of dormancy are the counterpart of those involved in metastatic outgrowth. 

Nowadays, DCCs cannot be detected by procedures commonly employed in routine clinical practice. For these reasons, cancer dormancy represents an outstanding challenge in the field of oncology and many scientists are gathering forces to understand its molecular underpinnings. The knowledge of mechanisms that drive dormancy may have relevant implications for future research in the field of medical oncology.

A large number of studies have investigated the key aspects of dormancy in BC [14,15,17] and explored different intrinsic and extrinsic mechanisms that could contribute to the disruption of dormancy, resulting in distant relapse [16,18,19,20,21,22]. At the cellular level, investigations revealed that different pathways cooperate with extreme complexity in regulating dormancy. Indeed, identification of genes that specifically regulate dormancy is extremely difficult [23,24]. In humans, only indirect observations suggest that DCC initiates metastasis in BC. For example, the presence of DCCs in the bone marrow of patients treated for early BC significantly correlates with reduced metastasis-free survival, as a milestone study showed [25]. In addition, DCCs of human BC can proliferate to form a mass when reinoculated into animal models, demonstrating their capacity for outgrowth [26]. Furthermore, when inoculated into mice, the outgrowth of BC stem cells with metastasizing capacity depends on the expression of specific factors in the host tissues, so the invasion of different sites is mainly related to the DCC adaptability to organ-specific niches [27]. 

Several authors have compared the biological features of DCCs to those of cancer stem cells (CSCs) [28], cells that are isolated from tumor mass by selection of cells with stem-like phenotypes. These cells are capable of generating tumor masses when inoculated into animal models. In addition, they are the putative reservoir of therapy-resistant cells that are responsible for progression after tumor response to anticancer treatments. Since they display these similarities with DCCs, some authors speculate that CSCs and DCCs are interchangeable concepts, although others consider DCCs different from CSCs, since the latter maintain slow-cycling potential to renew the cancer cell repertoire of tumors [29].

In recent years, epidemiological and clinical studies suggested a possible role in dormancy of diet, lifestyle and other host-related factors that have the capability to modulate systemic inflammation, thus prolonging the latency period of DCCs.

The aim of this review is to offer clinicians an overview of dormancy in breast cancer, guiding them in the basic understanding of the complexity that underlies this process and further focusing on the clinical and epidemiological factors that might influence its course over time.

## 2. Molecular Aspects of Dormancy

### 2.1. The Life Cycle of Dormant Cell

Dormancy commonly refers to a model that describes the behavior of specific types of cancer that tend to remain clinically silent between the surgical removal of the primary tumor and the subsequent progression and recurrence as metastatic disease. Two main hypotheses describe the genesis of recurrent disease. One postulates that isolated metastatic cells, known as disseminated cancer cells, survive in a state of permanent quiescence until disruption of dormancy occurs with subsequent proliferation that leads to the development of tumor masses. The other hypothesis suggests that small groups of metastatic cancer cells, also known as micrometastases, maintain an inner equilibrium between proliferation and death, remaining clinically silent [30]. In this review, we focus on the dormancy of isolated metastatic cells. 

DCCs reside in a specific environment, defined as niche, which regulates their life cycle in regard to space and time. The niche is a complex and mutable habitat in which interactions among different immune, connective and specific resident cells determine the fate of DCCs. Nevertheless, a DCC’s behavior also depends on its inherent genetic program that probably derives from cell-to-cell interplay in the primitive tumor. DCCs could dynamically interchange between different states during their life. The first step in the life cycle of DCCs is niche colonization with subsequent cell cycle arrest at the G0-G1 phase and adaptation to the organ-specific environment. Furthermore, the cell escapes immune surveillance through mechanisms of immune cloaking. Modifications of the niche ecosystem or of inherent cell programming could later induce proliferation and the onset of metastatic disease [29].

#### 2.1.1. Niche Colonization

**Figure 1:**
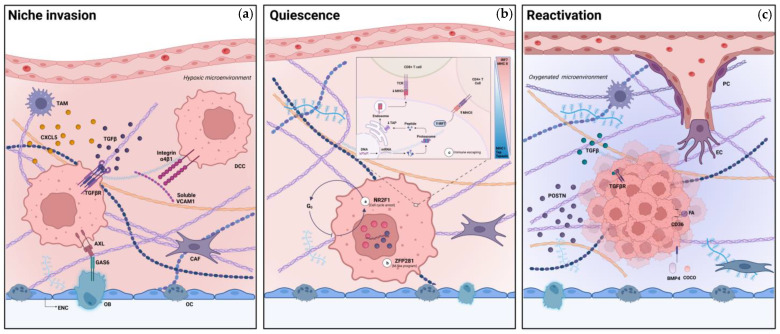
The life cycle of dormant cancer cells and their interactions with the niche ecosystem. An endosteal-perivascular niche is schematically represented above. The endosteal surface is placed below (cell lining below, ENC: endosteal cell or bone lining cell, OC: osteoclast, OB: osteoblast). Extracellular matrix (ECM) is the pink or blue space in the middle (pink: hypoxic environment, blue: oxygenated environment, fibrils: ECM protein structure, CAF: cancer-associated fibroblast, TAM: tumor-associated macrophage, DCC: dormant cancer cells). Blood vessels are above in the figure (PC: pericyte, EC: endothelial cell). (**a**) Niche invasion: DCCs anchor to ECM protein structure, establish a hypoxic microenvironment and interact with niche-resident cells that stimulate survival signals in the DCCs. (**b**) Quiescence: DCCs upregulate transcription factors that maintain a mesenchymal, quiescent phenotype and inhibit immune response with a direct interplay with T-lymphocytes and other immune-competent cells. (**c**) Reactivation: at some point, soluble factors secreted by the niche-resident cells and immune-competent cells and signals stimulated directly by DCCs induce angiogenesis and promitotic signals with subsequent reactivation of DCCs and manifestation as clinically detectable metastasis. (TCR: T-cell receptor, MHC: major histocompatibility complex, IRF7: interferon regulatory factor 7, TAP: transporter associated with antigen processing, CXCL5: C-X-C motif chemokine ligand 5, TGFβ: transforming growth factor β, GAS6: growth arrest-specific 6, NR2F1: nuclear receptor subfamily 2 group F member 1, ZFP281: zinc finger protein 281, POSTN: periostin, FA: fatty acid, BMP4: bone morphogenetic protein 4.

Once isolated, metastasizing cells reach the different niches and actively colonize the organ environment in order to enable the establishment of dormancy. The invasion and colonization of the different organ-specific habitats are influenced mainly by the organ of tumor origin, its histological subtype and by specific cell programming [31]. During the permanence of DCCs in the niche, hypoxia, soluble secreted hormones [12] and multiple receptor interactions of the cell surface elicit the inhibition of mitosis [32]. In particular, angiogenic and proinflammatory chemokines, such as C-X-C motif chemokine 5 (CXCL5), are putative mechanisms at the basis of niche colonization [33]. Transforming growth factor β (TGFβ), a chemokine with a wide spectrum of functions, plays a fundamental role in dormancy [34]. In the lung, TGFβ favors the colonization of the niche through the induction of angiopoietin-like 4, a mediator that disrupts lung microcirculation and helps cancer cells penetrate the tissue [35]. Conversely, in the endosteal niche, TGFβ increases the expression of factors involved in angiogenesis and favors the development of osteolytic metastasis [36], while osteoblast-secreted factors induce dormancy through the TAM family tyrosine-kinase receptors [37]. Finally, the molecular expression of specific genes that are involved in dormancy control might be common to different types of cancer, such as Axl, which encodes for the tyrosine-protein kinase receptor UFO (an enzyme of the TAM family) [38] and vascular cell adhesion molecule 1 (VCAM-1) [39] (Figure 1).

#### 2.1.2. Quiescence

Cell cycle arrest is crucial for the entry into a state of quiescence. Cell cycle arrest allows DCCs to persists after anticancer treatment and to escape immune surveillance. Certain transcription factors prominently act as promoters of survival and cell cycle arrest in DCCs [40,41]. Nonetheless, dormancy is not limited to the inhibition of cell proliferation induced by specific factors, but is a more complex condition, comparable to a peculiar embryonic state known as “diapause”. The latter defines a special behavior, which involves a network of different mechanisms of quiescence, especially focusing on reduced synthesis, stress response suppression, and specific interactions with the extracellular matrix (ECM). In addition, these mechanisms appear to depend on a unique cell programming system that confers chemo-resistance, supporting the hypothesis that the genesis of DCCs does not derive from pre-existing clones, but is related to a predetermined gene expression program [42]. Another hypothesis speculates that isolated metastatic cells can embrace a regenerative phenotype characterized by a particular inherent survival programming system that allows successful interactions with the niche to promote survival [43]. Indeed, epithelial-mesenchymal transition (EMT), which is the pivotal mechanism that initiates cell metastatization, seems crucial for the maintenance of a dormant phenotype, since mesenchymal-like programs lead to an increased invasive capacity and reduced proliferation. By contrast, epithelial-like programs revert the dormant state and induce metastatic outgrowth [41].

Immune escape is another essential mechanism to preserve the quiescent state of DCCs. The activity of transcription factor interferon-regulatory factor 7 (IRF7) prominently regulates cancer dormancy due to its substantial role in the inhibition of natural killer (NK) and CD8+ T-lymphocytes. In addition, CD4+ [44] and CD8+ T cells [45] promote quiescence by their active cytostatic effect on cancer cells. DCCs evade detection by CD4+ and CD8+ T-lymphocytes and NK as they reduce the expression of major histocompatibility complex class I (MHC-I), the transporter associated with antigen presentation (TAP) and tapasin, factors that are involved in the mechanisms of suppression of the “non-self” [46]. Moreover, IRF7 can stimulate the expression of MHC-II and elicit a phenomenon known as “immune cloaking”, through which a cancer cell can appear as a niche-resident immune cell [29]. Cancer cell-expressed MHC-II can also paradoxically inhibit lymphocytes [47] (Figure 1).

#### 2.1.3. Reactivation

DCCs need to revert their quiescent state to restore their full vitality and capability of proliferation in order to become a tumor mass. 

Firstly, the availability of nutritive macromolecules strictly conditions the viability of DCCs, so they necessarily decelerate their metabolism in order to prevent self-fatal damage caused by lack of nutrients [48]. In this regard, DCC reactivation and metastatic outgrowth seem to be related to CD36, a protein expressed by DCCs themselves, the activity of which is associated with dietary fat intake [49]. Furthermore, fat metabolism, as arachidonic acid-derived metabolites, could be involved in dormant cell reactivation [50]. 

The remodeling of the niche structure is another common mechanism that determines reactivation. The bone is an exquisite example, since its resorption represents an alteration of the endosteal niche equilibrium that is associated with an interruption of the dormant state [51].

A disruption of dormancy is frequent also when vasculogenesis occurs in the perivascular niche and the process is mediated mainly by periostin and TGFβ1 [52]. Conversely, in the lung niche, the TGFβ1 inhibitor Coco can block active inhibitory signals, thus leading to reactivation [53].

Besides, direct interaction between cancer cells and specialized niche cells leads to reactivation and progression to micrometastasis, as in the case of adherent junction mediation in the endosteal niche [54]. In addition, ECM regulates the state of dormant cancer cells, as changes in type III collagen composition could stimulate the reactivation of DCCs [55]. Moreover, the binding of collagen to the integrin receptor could activate the focal adhesion kinase (FAK)-Src-extracellular signal-regulated kinases (ERK) pathway that, in turn, stimulates mitosis. 

Furthermore, stromal Wingless/Integrated (Wnt) could reactivate dormant cells through stimulation of mitotic signals. Indeed, mouse models showed that autoinhibition of this signal by disseminated cancer cells induces quiescence and immune evasion in different organ niches [56] (Figure 1).

### 2.2. Immunity and Dormancy

The inhibition of the immune response and the use of mechanisms of immune cloaking (defined as the capability to evade immune detection) are primary characteristics of DCCs. A study showed that the bone marrow of patients who received surgery for early breast cancer was enriched with CD4+ and CD8+ memory T-cells, compared to healthy donors. Their levels correlated with the size of the primary tumor, as if the presence of tumor cells in the breast tissue could influence the immune equilibrium of the bone marrow [57].

Cancer has a known ability to induce immune suppression, as evidence shows that quiescent cancer cells in tumor masses create a hypoxic microenvironment that causes the exhaustion of T-lymphocytes [58]. Conversely, disseminated DCCs rely on different mechanisms, such as the limitation of antigen presentation by downregulation of MHC-I expression [59], through the activation of specific cellular responses, such as “unfolded protein response” [60]. Furthermore, specific subpopulations of regulatory T-lymphocytes (Tregs) that reside in the endosteal niche suppress the function of T-lymphocytes through adenosine-mediated pathways [61]. Similarly, the microenvironment of the perivascular niche is intrinsically immunosuppressive, due to the expression of immune checkpoints such as programmed death ligand 1 (PD-L1) [62]. 

CD4+ T-lymphocytes have a prominent role in dormancy since they are capable of downregulating vasculogenesis, thus prolonging quiescence [63]. In addition, evidence suggests that NK cells participate in the maintenance of dormancy through interferon γ (IFNγ) signaling. In the liver milieu, the expansion of activated hepatic stellate cells produces the depletion of NK cells through the secretion of CXCL12 and subsequent metastatic outgrowth [64]. Moreover, IFNγ signaling may be responsible for the cytostatic effect of CD8+ T-lymphocytes on DCCs [45]. IFN leads to the activation of specific signal transductions that regulate the transcription of genes related to various cellular responses. A recent study showed that primary breast cancer primes IFNγ-producing CD39+/PD-1+/CD8+ T-lymphocytes to promote mechanisms of lung immune dormancy. This was confirmed by the correlation between the presence of this specific subpopulation of T-lymphocytes and disease-free survival in breast cancer patients [65]. Interferon regulating factor 7 (IRF7) was shown to have a distinct role in bone metastasis since its elevated expression in primitive cancer cells correlated with bone metastasis-free survival in breast cancer patients. Indeed, IRF7 is involved in the active immune surveillance against dormant breast cancer cells (DBCCs) and, when restrained, metastatic outgrowth can appear [66]. Moreover, in tumors treated with chemotherapy, IRF7 pathway activation may promote modifications of the immune response in the tumor microenvironment with a transition from a suppressor cell- to a CD4+/CD8+ T cell-dominant response. IFNβ is the putative inducer since its increased serum level was associated with longer distant metastasis-free survival in breast cancer patients [67]. In general, IFN seems to be the final player in a number of pathways that regulate immune cell activity and induce growth arrest and senescence [68], thus representing a possible dormancy keynote [69].

Macrophages appear to be double-edged in respect to dormancy. Endosteal niche-resident macrophages may regulate the dormancy of disseminated breast cancer cells, conferring chemotherapy resistance and inducing cell cycle arrest through a direct interaction between surface proteins. On the other hand, the activation of the toll-like receptor 4 (TLR4) can inversely stimulate a switch of the macrophage phenotype into a proliferation-promoting cell, suggesting that inflammation may play a role in reverting the dormant state of DBCCs [70]. In this regard, neutrophils recalled in the lung tissue by inflammation may release proteases that initiate a dormant cell reactivation cascade [71,72]. Inhibitory cytokines, in particular, leukemia inhibitory factor (LIF), are known breast cancer metastasis suppressors [73]. The LIF receptor (LIFR), namely, prompts dormancy through a signal transducer and activator of transcription 3 (STAT3)-dependent signal in the endosteal niche [74]. Furthermore, interleukine-6 (IL-6)/STAT3 signaling can also limit the expression of immunogenic neoantigens, thus limiting the anticancer immune response [75]. Systemic inflammation that rampages after breast cancer surgery could represent another important factor in the regulation of the immunity of dormancy, as it can suppress a specific antitumor T-cell response [76].

## 3. Dormancy in Breast Cancer

In breast cancer, dormancy has been extensively studied since it is considered a distinctive trait of this disease. Mouse models showed that the dissemination of cancer cells might represent an early event in the development of breast cancer [10], especially for the hormone-sensitive subtype, which has an exquisite tropism for the bone. Several factors are involved in the homing and colonization of the endosteal niche. In particular, the increased expression of v-maf avian musculoaponeurotic fibrosarcoma oncogene homolog (MAF) [77] may promote events that favor the migration to the endosteal niche and its colonization. DBCCs increase their expression of chemokine receptors, such as CXCR4, and this boosts the homing to the endosteal niche where the respective ligand, CXCL12, is abundantly secreted by the niche cells [78]. Pathways related to the Src-AKT axis are also important in the survival response of the DBCC to CXCL12 [79]. 

The αvβ3 integrin expressed by DBCCs anchors them to ECM proteins of the bone, thus participating in the endosteal colonization [80]. Moreover, the upregulation of E-selectin ligands enhances the penetration of DBCCs in the perivascular niche of the bone marrow [81]. Furthermore, thrombospondin 1 (TSP-1) secreted by endothelial cells induces quiescence of the breast cancer cells in the perivascular niche [52]. This function may be related to the inhibition of vasculogenesis exerted by the dormant cells, since cell cycle arrest and the subsequent stress response, through the activation of autophagy [82], characterize their response to chronic hypoxia [83].

In the lung microenvironment, breast cancer cells interplay with alveolar type 1 cells and induce the production of ECM fibrils. Thereafter, signals mediated by the integrins-ECM interaction, as secreted frizzled-related protein 2 (SFRP2)-dependent signals, promote cell survival [84]. Nonetheless, DBCCs seem to remodel the lung niche with the secretion of TGFβ that stimulates the fibroblast to produce periostin. The latter maintains a stemness phenotype in DBCCs through the activation of Wnt signaling [27]. 

DBCCs that occupy the perivascular niche remain silent as long as vasculogenesis is inhibited. By contrast, neoangiogenesis, with the secretion of TGFβ-1 and periostin, encourages cells to exit from dormancy and promotes metastatic outgrowth [52]. Moreover, VCAM-1 can trigger a similar effect interacting with the α4β1 integrin [39]. 

A number of transcription factors are involved in the process of dormancy of breast cancer cells, from the induction of this complex state to its maintenance. Distinct transcription factors have prominent roles in dormancy. STAT3 may also participate in the dormancy of DBCCs, as its loss is associated with increased proliferation [74]. Furthermore, the nuclear factor kappa-light-chain-enhancer of activated B cells (NF-kB) induces stemness and a dormant phenotype in hormone-positive breast cancer cells [85]. Conversely, mitogen- and stress-activated kinase 1 (MSK1) is essential in the regulation of differentiation in luminal tumors; consequently, a lack of its expression is associated with reduced metastasis-free survival, since this protein is involved in the inhibition of metastasization [86]. Interestingly, evidence from clinical studies showed that patients with hormone-positive breast cancer have an increased risk of developing late metastatic recurrence [87].

## 4. Host-Related Factors, Inflammation and Breast Cancer Dormancy: From Biology to Clinical Suggestions

Dormancy has been widely studied in preclinical models, but few data have been collected in breast cancer patients, because it is a complex phenomenon, probably related to different processes, with a variety of molecular, supramolecular, genetic, epigenetic, hormonal and systemic regulatory pathways. Here, we report the study results and hypotheses that support the role of certain host-related factors in the dormancy process. All the mechanisms we aim to describe are involved, at different levels, in the regulation of systemic inflammation, which seems crucial in the natural history of cancer, based on current knowledge.

### 4.1. Inflammation and Breast Cancer Dormancy: General Aspects

Inflammation is defined as the overall response of an organism to an injury, through the activation of heterogeneous and complex pathways that aim to regulate and preserve tissue homeostasis. A body of preclinical evidence shows that inflammation may play a lead role in the biology of dormancy in breast cancer [88,89]. Inflammation and cancer seem closely related [90]. Indeed, during inflammatory processes, there is an increase in free radical levels as reactive oxygen and nitrogen species (RONS) bind DNA and induce mutations, leading to cellular instability, a state that favors carcinogenesis [91]. Moreover, the same molecules can trigger a series of pathways involved in further cancer outgrowth [92]. In addition, chronic inflammation could favor angiogenesis, a process in which cancer growth is supported by the supply of oxygen and nutrients through the genesis of new vessels [93]. 

One of the most studied processes involved in inflammation is prostaglandin production by Cyclo-oxygenases (COXs), enzymes that seem to have a prominent role in the regulation of immune surveillance and in the balance between DCCs and host. Cyclo-oxygenase-2 (COX-2), in particular, is an enzyme that catalyzes the production of prostaglandins from arachidonic acid, which could be released by cancer and microenvironmental cells. The activity of COXs is promoted by various growth factors, cytokines and chemokines, and its final effectors (prostaglandins, thromboxane, etc.) increase invasion, apoptotic resistance, angiogenesis and proliferation of tumor cells. This multitasking function implies an essential role in cancer evolution [94]. 

**Figure 2:**
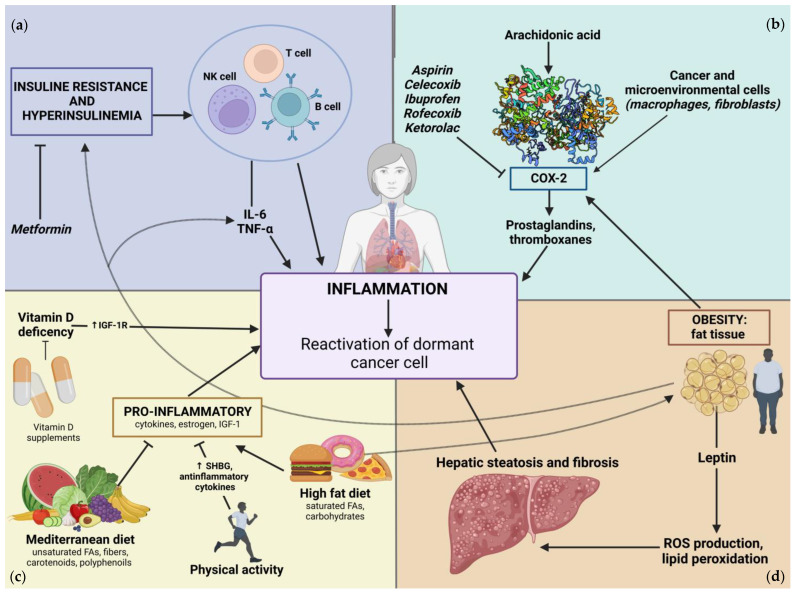
Host-related factors that influence the reactivation of DCCs through the modulation of systemic inflammation. (**a**) Insulin-resistance and hyperinsulinemia: elevated levels of insulin could affect the systemic inflammation, either directly, by activating immune cells, or indirectly, through the induction of proinflammatory cytokines (i.e., interleukin-6 (IL-6) and tumor necrosis factor α (TNFα) also secreted by adipose tissue) that, in turn, boost local inflammation. (**b**) Prostaglandin-mediated inflammation: cancerous and normal cells from neoplastic tissue could express cyclo-oxigenase-2 (COX-2) enzyme. It converts arachidonic acid into prostaglandins, thus inducing local inflammatory response and acting on vascular compartment. (**c**) Lifestyle: particular types of lifestyles could affect levels of pro- and anti-inflammatory cytokines. Mediterranean diet and physical activity have the potential to reduce the levels of systemic inflammation. Conversely, a high-fat/high-sugar diet directly stimulates the release of factors that sustain inflammation (as insulin-like growth factor-1 (IGF-1)) and increases the risk of obesity. Vitamin D deficiency correlates with increased IGF-1 receptor (IGF-1R) activity, further promoting inflammation. (**d**) Obesity: adipose tissue could actively secrete inflammatory cytokines and sustain systemic inflammation through leptin production, which foster radical oxygen species (ROS) formation and lipid peroxidation. These processes increase the risk of steatohepatitis, which exacerbates systemic inflammatory response. SHBG: sexual hormone binding globulin, T cell: T-lymphocyte, B-cell: B-lymphocyte, NK cell: natural killer cell.

Normal breast tissue is composed of hormone-sensitive epithelial cells, adipocytes and ECM, which is full of fibroblasts and macrophages, elements that are sensitive to inflammatory signals [95]. Additionally, in response to proinflammatory signals, adipocytes are able to secrete cytokines actively [96]. For this reason, there is a strong link between inflammation and adiposity, and this plays a clear role in cancer development. Beyond the mechanisms that underlie cancer biology, the epidemiological and clinical data reported in the last decades remain fundamental to understanding the possible role of inflammation in breast cancer dormancy. Evidence suggests that determined host-related factors implicated in the inflammatory response modulation could have a role in breast cancer dormancy. Figure 2 depicts their complex and intricate interplay.

### 4.2. Cyclo-Oxygenase-2 Activity and Anti-Inflammatory Drugs

COX-2 is inducible and overexpressed in inflamed tissue and cancer. COX-2 could represent a target for cancer treatment. Indeed, COX-2 inhibitors have been studied for their putative anticancer activity, as they could stimulate antiangiogenic, anti-inflammatory, and proapoptotic mechanisms [97], but data about their role in cancer treatment are still conflicting. Moreover, the risk–benefit profile of treatment with COX-2 inhibitors in this setting is still unknown since they present a risk of adverse cardiac effect.

In preclinical models, COX-2 was one of the genes associated with the development of brain metastasis from breast cancer [98].

Historically, a series of epidemiological studies conducted by Harris showed a preventive effect of nonsteroidal anti-inflammatory drugs (NSAIDs), such as aspirin, celecoxib and ibuprofen, in breast cancer development [99]. Later, Ashok conducted a case-control study in 2011 on more than 18,000 subjects, in order to evaluate the association between selective COX-2 inhibitor or nonspecific NSAID assumption and the risk of breast cancer development, and the authors reported that the lowest risk was associated with the use of a selective COX-2 inhibitor [100]. These data suggest a role for COX-2 in the carcinogenesis of breast cancer.

Regarding the interventional setting, the addition of a selective COX-2 inhibitor to the standard treatment of cancer patients in the palliative, adjuvant and neoadjuvant settings was evaluated in an important meta-analysis [97]. The study included considerable data deriving from 4516 studies and reported a limited overall benefit. Additionally, a study that tested the addition of celecoxib to adjuvant chemotherapy in triple-negative breast cancer patients showed a significant disease-free survival (DFS) benefit [101]. Unfortunately, the authors did not sub-analyze the distant metastasis-free survival (DMFS) outcome, which could be more informative on the eventual influence of COX-2 inhibitor on dormancy.

Alternatively, results of previous observational studies support the potential role of COX-2 in breast cancer dormancy. One of the most interesting studies analyzed tumor COX-2 expression in 1576 patients with treated localized BC and found a significant correlation between COX-2 expression and decreased DMFS [102]. Other authors reported similar results in smaller cohorts [103,104]. Another study reported a prolonged DFS with a non-significant association with local recurrence in a retrospective study of 570 localized BC patients compared to 52 healthy controls [105], highlighting the potential role of COX-2 expression in reducing the dormancy period of BC. Another retrospective study included 827 patients and showed that an intraoperative treatment with a single dose of a nonselective NSAID (ketorolac) in patients with elevated adiposity was associated with a decreased incidence of distant metastasis [106]. This stimulated the hypothesis that post-surgery inflammation could disrupt BC dormancy. In this regard, a large, multi-trial analysis that evaluated incidental cancers during five randomized controlled trials of daily aspirin showed a reduced incidence of metastasis in patients treated for localized BC [107]. 

Apart from these encouraging results, data from prospective trials on the use of COX-2 inhibitors to prolong BC dormancy remain conflicting. A randomized clinical trial of more than 2500 patients was conducted by Coombes in 2021 and recruited patients with epidermal growth factor-2 (HER-2)-negative early breast cancer, treated with adjuvant chemotherapy plus celecoxib, which was administered for 2 years, versus placebo. The primary endpoint, DFS, was not reached [108]. These findings were consistent with another study regarding the adjuvant setting that enrolled 1622 postmenopausal, hormone-positive patients who were receiving adjuvant aromatase inhibitors to associate either celecoxib or placebo. The addition of celecoxib did not improve DMFS in the final analysis [109].

In conclusion, COX-2 might have a role in breast cancer dormancy but its targeting with selective COX-2 inhibitors has not achieved the expected results, probably due to a pathway redundancy in the inflammatory cascades that limits the efficacy of a mono-target drug.

### 4.3. Obesity

Obesity is a multifactorial condition defined, according to the World Health Organization, as a BMI value over 30 kg/m^2^ [110]. Studies in obese mice demonstrated how diet-induced obesity produces a proinflammatory state with enhanced angiogenesis [111] and shorter recurrence-free survival in mouse models of BC [22]. Indeed, obesity seems associated with an increased level of neutrophils in lungs. In mice, obesity induced neutrophil infiltration in the lung tissue, and this was correlated with increased lung metastases development. In this investigation, weight loss could reverse this effect, decreasing the level of neutrophils in the lung. This supports the potential relevance of these findings in humans [112]. In addition, recent mouse models of BC showed that a high-fat diet increases the availability of palmitate in lung and liver niches; this could fuel metastatic outgrowth [113]. In humans, obesity was associated with late distant recurrence (5–10 years) after localized breast cancer treatment [114]. The obesity-specific risk of breast cancer is greater for the hormone-positive subtype [115], and this condition is associated with a worse prognosis and a reduced response to aromatase inhibitors in postmenopausal patients [116,117]. In this regard, several factors can affect the outcome. Leptin, a polypeptide mainly produced in fat tissue, has proinflammatory, proangiogenic, proinvasive and mitotic roles. In obese patients, increased levels of leptin, excessive reactive oxygen species (ROS) and increased levels of lipid peroxidation lead to a worsening of hepatic steatosis and fibrosis. Hepatosteatosis, a common condition in which fat accumulates in the hepatocytes, was studied as a risk factor for breast cancer recurrence in nonmetastatic breast cancer patients. A mono-institutional study, based on data collected from 422 patients, demonstrated that hepatosteatosis is correlated with increased late recurrence 5 years after the diagnosis, but the study lacked data regarding distant relapse [118]. Moreover, obesity stimulates the production of COX-2 by tissue macrophages, and this leads to an increased expression of preadipocyte aromatase and a subsequent increase in estradiol production. In addition, the serum of obese patients contains higher arachidonic acid levels than in normal-weight patients, thus potentially increasing prostaglandins production [119,120]. In a retrospective trial, Bowers investigated the outcomes of overweight/obese patients with estrogen-receptor positive BC exposed to NSAIDs during treatment with aromatase inhibitors. The results showed that overweight/obese patients had a higher risk of local or distant recurrence than normal-weight patients, and that exposure to NSAIDs led to a reduction in recurrence rate, especially in the postmenopausal population [121]. 

Being obese or overweight at diagnosis are conditions that play a role in cancer recurrence dynamics, especially in regard to the risk of developing detectable distant metastases over time. In a study that enrolled 777 patients with BC, the authors analyzed the temporal patterns of distant recurrence, discovering that overweight and obese patients tend to manifest distant relapse more quickly than normal-weight patients [122].

Previous observational studies suggested a possible correlation between the risk of distant metastasis and obesity in patients treated for localized BC [123,124]. A focus on the correlation between obesity and late recurrence was also the main object of a recent study which reported how lifestyle factors correlate with late recurrence in hormone-positive breast cancer patients. Obesity and a weight gain of more than 10% after diagnosis were associated with an increased risk of late recurrence, while physical activity was inversely associated with late mortality [125]. Although the study did not explicitly state the specific association with local or distant recurrence, late mortality could be considered as a surrogate of distant metastasis risk.

In conclusion, obesity represents a risk factor for the development and progression of breast cancer, and it may hamper the response to hormone therapy. In addition, studies suggest a correlation between adiposity and a shorter period of dormancy in BC. Therefore, the reduction in the imbalance of the production of proinflammatory cytokines in obese subjects, obtained by modulating their systemic metabolism, could improve the outcome of BC treatment, potentially prolonging the period of cancer dormancy (Figure 2).

### 4.4. Hyperinsulinemia and Insulin Resistance

Obesity can lead to an imbalance between insulin production and tissue resistance. Insulin is a peptide, secreted by pancreatic β-cells, which exerts metabolic functions on the liver, skeletal muscles and white adipose tissue. This hormone leads to an increase in glucose uptake by skeletal muscles. In the adipose tissue, it enhances fatty acid and glucose uptake, while in the liver it stimulates glucose metabolism and glycogen storage. Insulin resistance is defined as a condition in which peripheral tissues are less sensitive to insulin activity. Chronic insulin resistance leads to an increase in insulin production and in its bloodstream release which, in order to maintain normal glucose levels, causes a condition named hyperinsulinemia [126]. Preclinical and clinical studies showed that hyperinsulinemia correlates with inflammation, and that both these factors increase the release of cytokines, such as IL-6 and tumor necrosis factor α (TNF-α), in the adipose tissue [127], eliciting a direct activity on immune system cells, in particular, NK and T- and B-lymphocytes [128].

Hyperinsulinemia is associated with a higher risk of breast cancer, as demonstrated in a case-cohort study by Gunter in 2015, in which the authors showed that insulin levels were positively associated with breast cancer development in postmenopausal women [129]. Lawlor reported similar results in a population of elderly patients [130]. Notably, high levels of insulinemia are associated with a global increase in cancer mortality [131,132].

Based on this evidence, many clinical studies have attempted to demonstrate the role of metformin in breast cancer. Metformin is a biguanide commonly employed in the treatment of diabetes. It improves peripheral insulin sensitivity, leading to the reduction of hepatic glycogenesis and an increase in glucose uptake. Interestingly, metformin may also have a potential anticancer role, acting both directly on cancer cells and, indirectly, through the reduction of insulin and insulin-like growth factor 1 (IGF-1) levels. 

In the literature, studies on the role of metformin in preventing breast cancer have yielded conflicting results. A meta-analysis of Yang, which included 15 case-control and retrospective cohort studies, aimed to understand the relationship between metformin and breast cancer incidence and mortality. Unfortunately, the intake of metformin did not improve clinical outcomes [133].

Conversely, a recent trial, known as the “Sister Study”, attempted to analyze in depth the role of metformin in a population at risk for breast cancer. A population of 50884 subjects with a significant family history of BC, regardless of BRCA status, was enrolled. Information about the use of metformin or other antidiabetic drugs was collected. Among patients with type 2 diabetes, the use of metformin did not correlate with the incidence of breast cancer. In this population, the use of metformin was associated with a reduction in the incidence of luminal breast cancer, suggesting an association with sex hormone activity [134]. 

Regarding breast cancer treatment, Goodwin designed the MA.32 study, a Phase III, placebo-controlled, double-blind trial in which 3649 early breast cancer patients randomly received standard adjuvant therapy plus metformin or placebo. The study did not achieve its primary endpoint, as treatment with metformin did not improve disease-free survival rates [135]. Similar results were obtained at MD Anderson in a single-institution, case-control study which enrolled women with triple-negative breast cancer, as they also could not demonstrate an impact of metformin in the adjuvant setting [136].

Data regarding the neoadjuvant setting seem more encouraging. An observational study, conducted on 291 diabetic patients, demonstrated that the intake of metformin was associated with a higher chance of pathological complete response (pCR) compared to other antidiabetic therapies [137]. In a nondiabetic population, the METTEN study demonstrated an improvement in pCR by adding metformin to neoadjuvant chemotherapy vs. chemotherapy alone in HER2-positive BC patients. Unfortunately, the study was prematurely discontinued because of slow accrual, so the results did not reach statistical significance because of the small sample size [138].

Finally, despite the potential effect of insulin signaling in systemic inflammation, current clinical evidence about the role of antidiabetic drug administration in the regulation of BC dormancy seems discouraging. In this regard, a better knowledge of the role of insulin metabolism in dormancy regulation could bring improvements to the therapeutic approach (Figure 2).

### 4.5. Diet, Nutrition, and Supplementation

Diet could influence the production of proinflammatory cytokines, suggesting a role in the regulation of cancer metabolism. Certain nutrients, such as saturated fatty acids and carbohydrates, increase the production of proinflammatory cytokines [139]. 

Particularly, epidemiological and preclinical data suggest that red meat and saturated fats increase levels of proinflammatory cytokines, IGF-1 and estrogens, ultimately increasing the risk of breast cancer [140]. Saturated fats can affect intracellular pathways related to carcinogenesis, so a high saturated-fat diet could be associated with an increased risk of breast cancer, especially for the hormone-positive subtype [141,142].

On the other hand, high polyphenol intake might reduce breast cancer incidence and recurrence: vegetables, fruit, fish and whole grain may reduce inflammation [143] and the Mediterranean diet reduces the levels of proinflammatory markers [144].

Currently, vitamin D is becoming a topic of interest in different fields, in particular, in carcinogenesis. A study suggests that vitamin D deficiency could lead to a higher risk of recurrence in breast cancer patients [145]. The underlying mechanism is not completely understood and is probably linked to an upregulation of the IGF-1 receptor (IGF-1R), a receptor involved in a downstream pathway that prevents apoptosis and increases cell survival [146,147]. This pathway has a relevant clinical role, as IGF-1R overexpression correlates with a worse prognosis in breast cancer [147]. Vitamin D supplementation can downregulate IGF-1R expression and should be considered as a possible key point of intervention [146].

Low magnesium levels are involved in oxide nitric production, subsequent vascular-endothelial growth factor (VEGF) production and neoangiogenesis [148], but conflicting data are found in the literature about magnesium supplementation in breast cancer and its clinical significance. Preclinical data on magnesium and cancer show a possible negative effect on cell implantation in metastatic sites [149].

Based on these data, additional studies were conducted in order to understand the role of diet in patients with breast cancer. 

To standardize the inflammatory potential of diet, the diet-inflammatory index (DII) was validated. This tool was created by analyzing the role of 45 foods and nutrients on some specific inflammatory markers (IL-1, IL-6, TNFα, IL-10, C-reactive protein), and a score from −1 to +1 was assigned to each item based on its capacity to change inflammatory marker levels [150].

Several studies investigated the role of DII in breast cancer patients. Tabung prospectively analyzed 7495 postmenopausal breast cancer patients and found that patients with a proinflammatory diet before diagnosis were more likely to be obese and overweight, usually had a lower level of education and were less likely to engage in physical activity. In this study, DII did not correlate with the risk of breast cancer, but an association between this dietary pattern and breast cancer mortality was found [151]. Another study collected data from about 530 patients who underwent surgery for early breast cancer and found that DII was associated with reduced mortality [152].

In 2006, Chlebowski published the Women’s Intervention Nutrition Study (WINS), to understand whether a dietary intervention could prolong relapse-free survival in women with breast cancer. A total of 2437 women were enrolled: 975 of them underwent dietary interventions, mainly in the form of reducing fat intake, and 1462 were included in the control arm, in which a free diet was permitted with minimal dietary counselling. After a 60 month follow-up, no difference in overall survival was detected, but women in the dietary intervention group had a 24% lower risk of relapse than those in the control group (HR = 0.76; 95% CI = 0.60 to 0.98) [153]. 

Particular diets seem to have a role in reducing recurrence risk and improving mortality. The hypothesis explores the potential effect of this type of diet in lowering systemic inflammation [154]. The reduction in recurrence risk and mortality, together with the potential anti-inflammatory effect, suggest a possible role of diet in prolonging dormancy or in preventing the reactivation of DCCs.

### 4.6. Physical Activity

Physical activity is associated with a reduction in the risk of breast cancer, especially for postmenopausal women [155]. The hypothetic mechanism deals with weight loss in relation to hormone and inflammation levels. Regular activity leads to the expression of anti-inflammatory cytokines and decreases the levels of proinflammatory mediators [156]. Moreover, regular physical activity is associated with an improvement in insulin sensitivity with lower levels of IGF-1 [157].

A recent, interesting paper by Lynch explains how the lack of physical activity is linked to potential carcinogenetic mechanisms [158]. In addition, physical activity may be associated with reduced mortality in localized or locally advanced BC patients [159]. From a clinical point of view, current NCCN guidelines on breast cancer recommend certain levels of physical activity in order to improve clinical outcomes and quality of life [160]. This evidence derives from clinical studies that investigated the role of exercise during adjuvant treatment, although there were no statistically significant effects on disease-free survival [161,162] and relapse-free period. In addition, a large US study demonstrated a reduction in breast cancer mortality in subjects who performed certain levels of physical activity [163]. Similarly, a Norwegian study found that post-diagnosis physical activity levels were associated with a significant trend in decreased breast cancer-specific mortality, independently of body mass index [164] (Figure 2).

### 4.7. Inflammation and Breast Cancer Dormancy: Conclusions

Despite suggestions of the possible role of inflammation in dormancy, clinical validation lacked evidence. The amount of preclinical and clinical studies has rapidly increased over time, as interest has increased recently. Inflammation has a known role in many diseases, but the targeting of specific pathways in cancer patients did not produce significant benefits, probably due to the extreme complexity of the biological mechanisms involved. Lifestyle interventions appear to constitute a reasonable approach to obtaining a more comprehensive effect on inflammation pathways, combatting drug toxicities.

## 5. Discussion

Dormancy is a complex biological behavior of metastatic cancer cells sustained by specific cellular programming and mediated by their interactions during their residency in the niche microenvironment, through several cross-talking pathways that include inflammatory, pro-angiogenic and immune regulations. The interaction between cancer and host, modulated by intrinsic and extrinsic factors, regulates the dynamic of quiescence and reactivation. Particularly, the dormancy of BC is a determinant for patient prognosis. Clinical information about dormancy may be crucial to understanding cancer behavior, especially in the cases of late recurrence, a frequent event in hormone-positive breast cancer that additionally generates tremendous anxiety in patients.

Unfortunately, despite a great number of preclinical models and studies about the biological factors involved in dormancy, therapeutic application of this knowledge is currently difficult. Starting from preclinical studies, many authors attempted to demonstrate a correlation between factors that biologically influence the dormancy process and patient prognosis, through common clinical outcomes (DFS, OS, breast cancer-specific mortality).

One of the most studied mechanisms that may affect BC dormancy is inflammation, and epidemiological studies confirmed the assumption that anti-inflammatory drugs may be associated to better prognosis in breast cancer. This approach was explored in randomized clinical trials in neoadjuvant, adjuvant and metastatic settings. The administration of COX-2 inhibitors, added to standard therapy, demonstrated a slight benefit in breast cancer patients, but data remain conflicting.

Furthermore, reducing inflammation levels by the use of specific drugs is a reasonable approach, but the magnitude of its benefit is not clear to date, and reflection on the risk–benefit profile of the assumption of COX-2 inhibitors have to be considered.

Another possible manner to target the inflammation axis is to operate upon modifiable host-related factors, such as obesity, insulin resistance, diet and physical activity.

To date, obese and overweight breast cancer patients seem to have a worse prognosis, especially in the case of the hormone-positive subtype. Regarding this, physical activity could induce weight loss, playing a role in decreasing breast cancer-specific mortality. In addition, hyperinsulinemia is a known risk factor for breast cancer and it is associated with an increase in cancer-specific mortality. Again, the release by fat tissue of proinflammatory factors seems to be the key point in this case, further increased when hyperinsulinemia coexists. The attempt to decrease insulin levels by administering metformin was investigated in different clinical trials, but the results obtained were inconclusive.

Lastly, diet has a role in the production of proinflammatory cytokines. Even if there are no clear data on the clinical impact of single nutrients, some foods may reduce levels of systemic inflammation.

In conclusion, despite the relevance of inflammation and immunity in the dormancy process in preclinical models, therapeutic approaches based on these principles lack meaningful results to date. Studies could barely demonstrate a linear correlation between the molecular and the clinical levels in terms of patient outcomes (DFS, OS), either for the redundancy of the pathways involved in dormancy or for the high number of patients who have to be enrolled for significant events to be observed.

Currently, several standard therapeutic approaches could have significant effects on dormancy. In the bone tissue, bone resorption mediated by osteoclasts is activated upon the release of TGFβ, which could also stimulate tumor outgrowth. Bisphosphonates or receptor activator of nuclear factor kappa beta (RANK) inhibitors may reduce the awakening of DCCs through blockade of bone resorption [165,166]. In addition, cyclin-dependent kinases 4 and 6 (CDK4/6) inhibitors, small molecules that represent the current standard therapy, in association with hormone therapy, for the adjuvant treatment of high-risk, hormone-positive BC, may prolong cancer cell dormancy by preventing the transition from G1 to S phase [167].

On the other hand, metronomic chemotherapy, defined as the continuous and regular administration of low-dose chemotherapy for long periods [168], might inhibit angiogenesis [169,170], thus suppressing DCC reactivation.

Behavioral interventions that may modulate systemic inflammation were investigated with encouraging results in breast cancer patients, suggesting that certain habits may influence dormancy through the modulation of immunity and niche microenvironment. Indeed, international guidelines suggest recommending that patients pursue a healthy lifestyle [160].

Nowadays, interest in targeting the dormancy process in breast cancer is progressively increasing. Table 1 summarizes a list of ongoing clinical trials dealing with specific drugs or interventions targeting the dormancy process (available at https://clinicaltrial.gov (accessed on 18 February 2023)).

## 6. Conclusions and Future Directions

A deep understanding of the dormancy process presents the opportunity to design clinical trials in perioperative, neoadjuvant and adjuvant settings.

Future observational studies should aim to unravel the role of systemic and local tumor inflammation in dormancy. Studies should evaluate the levels of circulating inflammatory cytokines in the relapse-free period to identify specific regulators of the dormancy process. Otherwise, studies that employs longitudinal bone marrow aspiration may be less feasible but much more informative in this context, as they provide the possibility for the early detection of DCCs and to develop preclinical models in order to investigate the distinctive traits of the niche microenvironment.

Clinical trials should evaluate the effect of inflammation-targeted interventions in the (neo) adjuvant setting. The combination of antiblastic treatment and lifestyle intervention could be effective, taking into consideration that such studies could be arduous to conduct. The combination of antiblastic treatments with drugs that can modulate systemic inflammation could be more feasible, but with a higher risk of adverse outcomes. Additionally, in the perioperative setting, the use of anti-inflammatory therapies to treat surgery-related pain is widely diffused; thus, conducting studies that evaluate the effect of specific anti-inflammatory drugs or immune-modulators on breast cancer dormancy should be feasible.

Finally, in the future, public health initiatives should raise consciousness about the crucial role of a healthy lifestyle intervention to obtain positive effects for a long-term period.

## Figures and Tables

**Table 1:** List of ongoing trials with a dormancy-based rationale.

Number Nct	Intervention	Target	Status	Setting
NCT04841148	Hydrossicloroquine, or Avelumab, With or without Palbociclib	Autophagy PD-L1 CDK4/6	Recruiting	Early BC, after adjuvant treatment
NCT03774472	Hydroxycloroquine, Palbociclib and Letrozole	Autophagy CDK4/6	Active, not recruiting	Early BC, before surgery
NCT04523857	Abemaciclib with or without Hydroxicloroquine	Autophagy CDK4/6	Recruiting	Early BC after surgery
NCT05550415	Simvastatin	EMT	Recruiting	Early TNBC in neoadjuvant treatment
NCT02876302	Ruxolinitinib	IL6/JAK/Stat pathway	Active, not recruiting	Preoperative Inflammatory BC
NCT0190504	Metformin		Active, not recruiting	Women at risk for BC
NCT02928978	Ruxolinitinib	IL6/JAK/Stat pathway	Recruiting	Women with premalignant breast biopsy
NCT04267796	Lifestyle interventions	Inflammation	Recruiting	Women with normal Body Mass Index (BMI)
NCT02235051	Lifestyle interventions	Inflammation	Active, not recruiting	BC survivors
NCT04965246	Physical Exercise	Inflammation	Recruiting	Obese BC survivors
NCT02927249	Aspirin	Inflammation	Active, not recruiting	Stage II-III BC after treatment
NCT03454529	Simvastatin	EMT	Active, not recruiting	Stage I-IIb BC
NCT04711109	Denosumab	RANK/RANKL	Recruiting	Women with BRCA1 germline mutation
NCT02750826	Lifestyle intervention	Inflammation	Active, not recruiting	Obese and overweight BC survivors
NCT04542135	Sulindac	Inflammation	Recruiting	Women at risk for BC

## Data Availability

Not applicable.
